# Supplementary material for: Realization of sextuple polarization states and interstate switching in antiferroelectric CuInP2S6
Source: Nat Commun. 2024 Mar 26;15:2653. doi: 10.1038/s41467-024-46891-8 (PMC10965965; doi:10.1038/s41467-024-46891-8)
Supplement: Supplementary file 1 — Supplementary Information [file 41467_2024_46891_MOESM1_ESM.pdf]

Supplementary Materials for

Realization of sextuple polarization states and interstate switching in antiferroelectric $CuInP_2S_6$

Tao Li[1,6], Yongyi Wu[1,6], Guoliang Yu[2], Shengxian Li[2], Yifeng Ren[3], Yadong Liu[1], Jiarui Liu[1], Hao Feng[1], Yu Deng[3], Mingxing Chen[2,4]*, Zhenyu Zhang[5]*, Tai Min[1]*

[1]Centre for Spintronics and Quantum Systems, State Key Laboratory for Mechanical Behavior of Materials, School of Materials Science and Engineering, Xi'an Jiaotong University, Xi'an 710049, China

[2]Key Laboratory for Matter Microstructure and Function of Hunan Province, Key Laboratory of Low-Dimensional Quantum Structures and Quantum Control of Ministry of Education, Synergetic Innovation Centre for Quantum Effects and Applications (SICQEA), School of Physics and Electronics, Hunan Normal University, Changsha 410081, China

[3]Solid State Microstructure National Key Lab and Collaborative Innovation Centre of Advanced Microstructures, Nanjing University, Nanjing 210093, China

[4]State Key Laboratory of Powder Metallurgy, Central South University, Changsha 410083, China

[5]International Center for Quantum Design of Functional Materials (ICQD) and Hefei National Laboratory, University of Science and Technology of China, Hefei, Anhui 230026, China

[6]These authors contributed equally to this work.

*Corresponding authors: Mingxing Chen, mxchen@hunnu.edu.cn; Zhenyu Zhang, zhangzy@ustc.edu.cn; Tai Min, tai.min@xjtu.edu.cn.

Supplementary Note 1: Determination of monolayer number in a domain block

To rationalize the number of MLs and their AFE/FE configuration in a DB, we conducted further calculations of 4-ML and 6-ML CIPS in addition to the 1-, 2- and 3-ML reported in the AFE/FE model. We found that the ground state of CIPS films spanning from 2- to 6-ML all exhibit a net AFE state (undiscernible by PFM) with the top and bottom MLs in FE state that are always arranged with head-to-head AFE coupling, and the middle MLs are all in AFE state (Fig. S10). Furthermore, the 2- to 6-ML CIPS films also share several consistent key features in their polarization configurations. First, for the energy-favorable arrangements of the films, the head-to-head AFE coupling between FE layers is always an energy-efficient configuration. Taking 6-ML CIPS as an example, complete AFE MLs show total energy increased by about 2~5 times compared to head-to-head configurations, and the tail-to-tail configuration of the FE MLs has total energy increased by about 7~19 times (Fig. S12). Secondly, the middle MLs within 3-, 4-, and 6-ML CIPS films can be reversibly switched between FE and AFE states by appropriate electric field excitation, leading to the possible metastable states ($\leq$ 55 meV) with the mixed FE and AFE MLs in CIPS films (Fig. S12). Thirdly, as mentioned before, there is an energetically favorable intermediate AFE state in the kinetic pathway during the switching of the FE orderings under a relatively small reversed E-field (Fig. S13). We can reasonably infer that these critical features still persist in our DBs comprised of more than 6 MLs. Experimentally, the thinnest CIPS film showing double polarization states had a thickness of 12-ML. It is widely recognized that two polarization states can be considered as a result of the complete reversal of a domain, which can be readily regarded as a single DB. Excluding the two outermost head-to-head FE MLs, we would expect a single DB to consist of about 10 MLs in the 12-ML CIPS. Our thinnest CIPS film with quadruple polarization states had a thickness of 20 MLs, thus, we would anticipate about 9 MLs for each of the two DBs. For the sextuple polarization states, we found the thinnest CIPS film was about 35-ML thick so far, and using a 10-ML DB, there would be three additional MLs. We assume those three extra MLs may exist between the DBs as disordered or AFE MLs, and do not contribute to the total polarization during the switching process.

Supplementary Note 2: Reversible switching of sextuple polarization states via the hybrid mechanism

As shown in Fig. 4b, taking S-1 (LH-1) as the starting state of the hysteresis loop, transitioning from S-1 to S-2 occurs at relatively small negative E-fields (-z), which induces regular polarization switching from up to down (path-A in Fig. S14) of all MLs in the top and middle DBs. Because of the weaker field in the bottom DB, the MLs closer to the tip are switched to an intermediate AFE state via the aforementioned half-layer-by-half-layer switching mechanism (path-B1 in Fig. S14) as described in the AFE/FE model (see Supplementary Note 1), while the rest AFE MLs remain unchanged. The net polarization from the sum of the three DBs has a slightly reduced value for S-2 (LH-2) due to the appearance of more AFE MLs in the bottom DB. The transition from S-2 to S-3 occurs in a strong negative E-field range, and Cu ions can migrate across the vdW gaps at a sufficiently large field (path-C in Fig. S14), resulting in a polarization direction opposite to the applied E-field[1,2]. In this case, the top DB, exposed to the strongest E-field, experiences ion migration in all FE MLs resulting in upward polarization, while half of the middle DB exhibits Cu ion migration where the E-field is strong enough, and the rest remains downward polarization. The bottom DB experiences the weakest E-field, insufficient to cause any Cu ion migration, but the top few MLs can be switched from AFE state to downward FE state again via the half-layer-by-half-layer switching (path-B2 in Fig. S14) aligned with the applied E-field. The rest MLs in the bottom DB that are furthest from the tip still remain as AFE states. Thus, the polarization value for S-3 is largely reduced mainly due to the canceled upward and downward polarizations in the middle DB and has an effective direction opposite to the applied E-field (LL-1), again consistent with the experimental findings. Subsequently, the applied E-fields gradually decrease from the negative maximum to zero causing the transition from S-3 to S-4. Under this E-field strength, all MLs in the top and middle DBs undergo a regular polarization switching from up to down along the E-field direction, while the bottom DB remains unchanged with the top few MLs also along with the E-field. This configuration of three DBs results in an increased net polarization value back to LH-1 (S-4), with the same magnitude but opposite direction to S-1. Next, the transition from S-4 to S-5 occurs under small positive E-fields (+z) following the same rules applied for the S-1 to S-2 transition, resulting in an upward net polarization for S-5 (LH-2), with the same magnitude but opposite direction to S-2. When the positive E-field is further increased to a sufficient value, Cu ion migration crossing the vdW gap can be triggered again, similar to what occurs in S-3. However, it is noteworthy that the V_c for S-6 always demonstrated a higher value than that of S-3 (Fig. 4b). Consequently, the E-field penetrates deeper into the CIPS film causing more MLs to exhibit Cu ion migration, switching all MLs in the top DB and most of the MLs in the middle DB to FE state in the direction opposite to the E-field, while the rest few MLs in the middle DB remain in an upward direction aligned with the E-field. The bottom DB in this case has the same type of behavior as that for S-3. As a result, S-6 (LL-2) has a larger net polarization compared to S-3 (LL-1), and both have an effective direction against the applied E-field. As the poling E-field is gradually reduced from the positive maximum to zero, S-6 undergoes a transformation to S-1 in a manner similar to the transition from S-3 to S-4, completing the full switching cycle. A probable configuration of three DBs for the mutually switchable transitions from S-1 to S-6 is illustrated in Fig. S15, from which the ratios

between each polarization level are in line with those between piezoresponse levels observed experimentally.

Supplementary Note 3: Impact of tip size on electric field distribution and domain formation

We conducted additional simulations to model the nonuniform electric field distribution under a 5 nm tip using COMSOL Multiphysics (Fig. S21). When compared to the scenario with a 25 nm tip radius, at the same field strength (e.g. 10^7 V/m, close to the coercive field of CIPS reported in the literature), the field extended distance along both in-plane (x-axis) and out-of-plane (z-axis) directions is shrunk to half. In other words, the active volume of CIPS under the 5 nm conductive tip is reduced to about 1/6 of that using a 25 nm tip. In addition to the largely shrunk excited volume, the sensible range of the tip is also significantly reduced with the tip radius.

To further investigate the above-mentioned field-gradient effect, we have performed PFM experimental measurements on both a classic ferroelectric ceramic (PMN-PT single crystal) and a CIPS film using a ~5 nm radius tip (AD-2.8-SS, Adama, Ireland), which has a conductive diamond coating with similar stiffness and resonant frequency as those of the ~25 nm tip (PPP-EFM, Nanosensors, Switzerland) (Fig. S22). The ~25 nm tip can detect reliable polarization switching for both materials. Unfortunately, it turns out that polarization switching was not achieved even in the classic ferroelectric ceramic using the ~5 nm tip in our current system. We will continue to investigate the field gradient effect on the intrinsic multiple polarization orders in CIPS systems, and we speculate that, for a trilayer system, the 5-nm tip should be operative in defining multiple polarization states.

Sample	Cu/ At%	In/ At%	P/ At%	S/ At%	Cu/In
Stoichiometric	10	10	20	60	1
1	11.14	11.73	19.58	57.54	0.95
2	10.97	11.39	19.63	58.00	0.96
3	10.93	11.02	19.87	58.17	0.99
4	10.94	11.11	19.95	58.00	0.98
Average	11.00	11.31	19.76	57.93	0.97
Standard deviation	0.10	0.32	0.18	0.27	0.02

Table S1 | Compositional measurements over different locations of CIPS crystal by EDS. Our CIPS crystals have the composition of $Cu_{1.14\pm0.01}In_{1.17\pm0.03}P_{2.05\pm0.2}S_{6.00\pm0.03}$, where Cu/In ratio is close to the stoichiometric value.

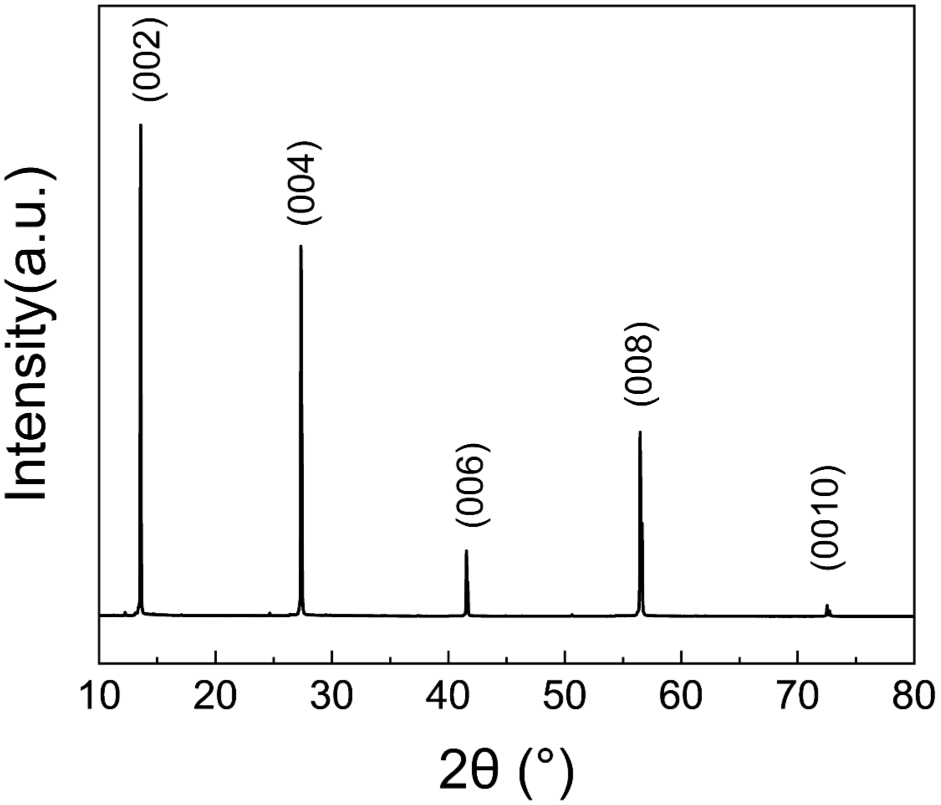

Fig. S1 | XRD spectrum of our CVT-grown CIPS crystal observed at room temperature. Only peaks along the c-axis were identified, which indicated that it is the single crystal CIPS. The c-lattice value was calculated to be 13.04 Å, close to the value (13.00 Å) reported by Deng et al. showing a trigonal crystal structure[3] (Fig. S2).

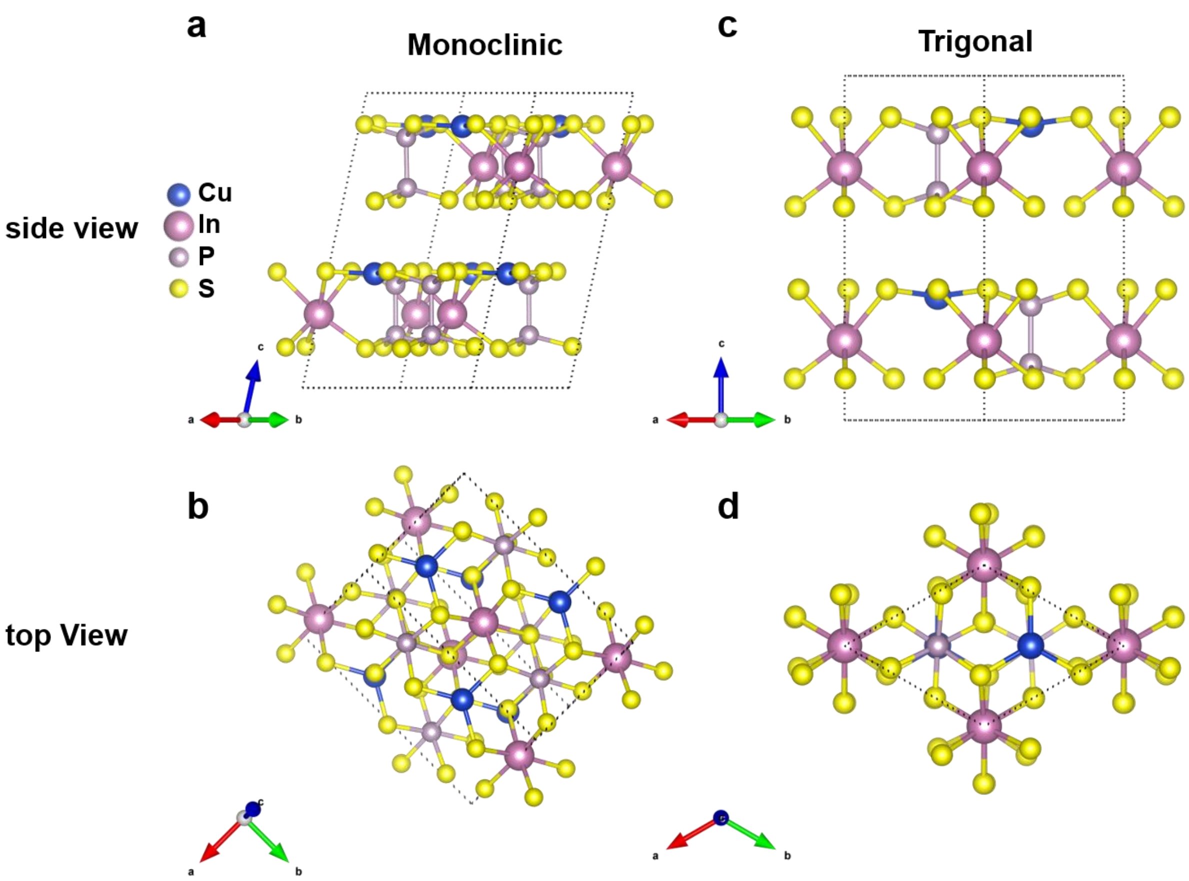

Fig. S2 | Illustration of monoclinic structure (*Cc* space group) and trigonal structure (*P31c* space group) of CIPS crystal. **a,** The cross-sectional view of the monoclinic structure. **b,** The top view of the monoclinic structure. **c,** The cross-sectional view of the trigonal structure. **d,** The top view of the trigonal structure. The unit cell of each structure is enclosed by dotted grey lines. The lattice parameters of the monoclinic structure are a=6.09 Å, b=10.56 Å, and c=13.17 Å, respectively[4], while the lattice parameters of the trigonal structure are a=6.09 Å, b=6.09 Å, and c=13.00 Å, respectively[3].

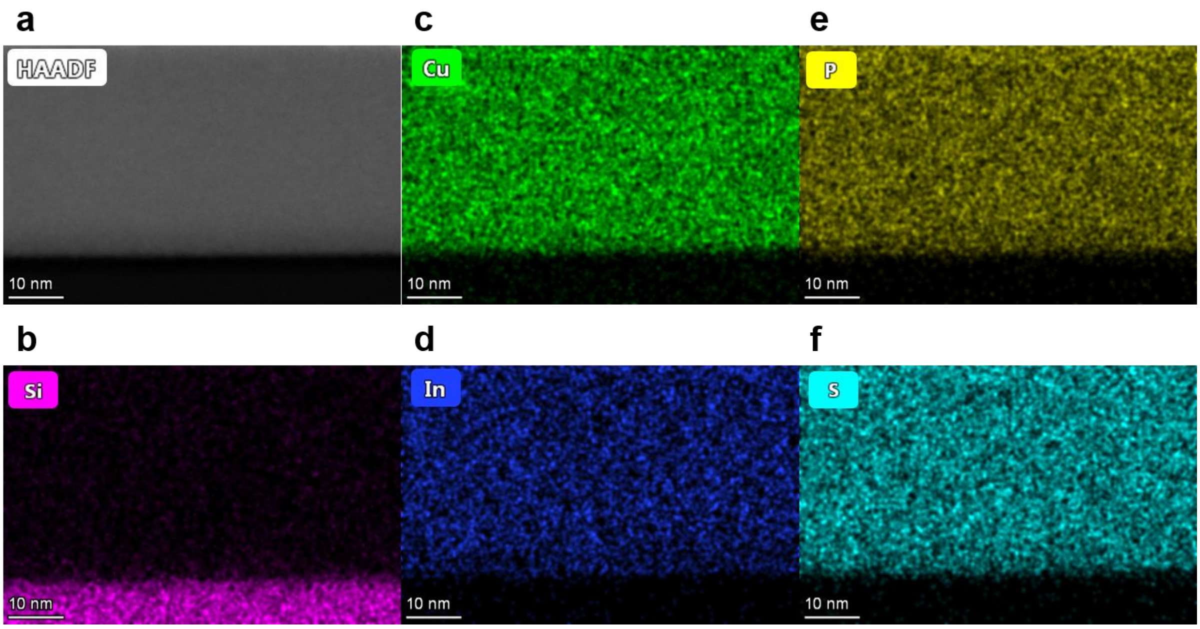

Fig. S3 | Element distribution of cross-sectional CIPS film exfoliated on n-type Si substrate by HR-TEM. **a,** The corresponding morphology image. **b,** The elemental distribution of Si, dominated at the substrate region. **c,** The elemental distribution of Cu. **d,** The elemental distribution of In. **e,** The elemental distribution of P. **f,** The elemental distribution of S. The distribution of the four elements (Cu, In, P, and S) was uniform without phase separation, no IPS phase was identified in our CIPS films. The spatial resolution is about ~0.8 Å.

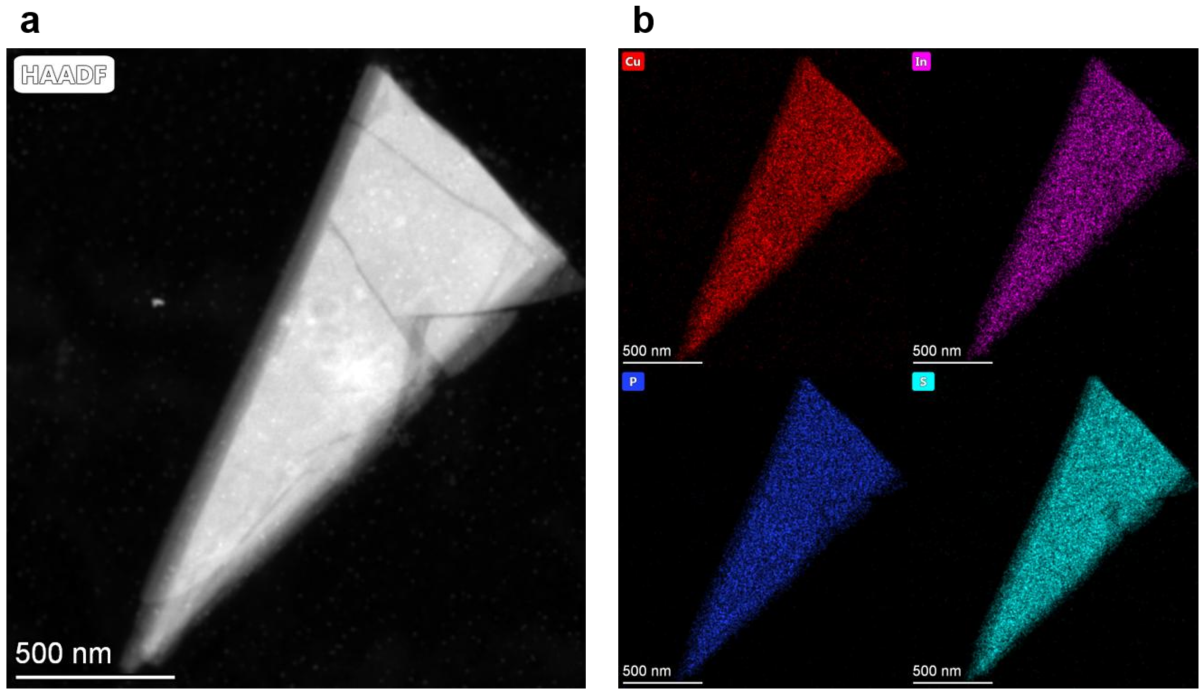

Fig. S4 | Element distribution of in-plane CIPS film (~40 nm thickness) by HR-TEM. **a,** The corresponding morphology image from the top view. **b,** Elemental distribution of Cu, In, P, and S in this CIPS film. The distribution of the four elements (Cu, In, P, and S) was still uniform without phase separation in the entire flake area.

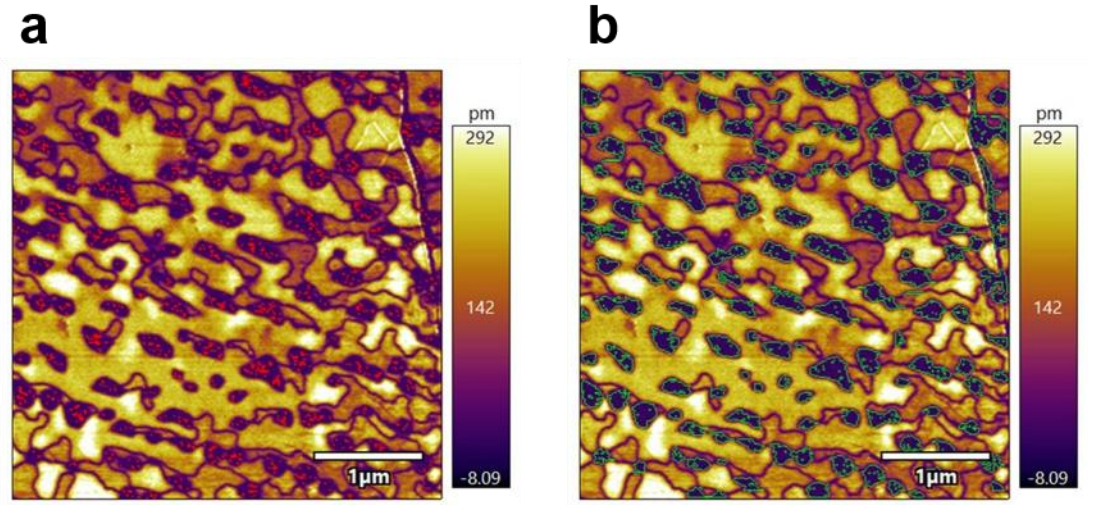

Fig. S5 | Amplitude images demonstrating the different levels of response strength. a, The region with the raw amplitude signal strength < 30 pm (system noise level) is marked in red color. The image has 65536 (256 × 256) pixels in total. The marked regions take 1242 pixels, which occupy about 1.9% area of the entire image. **b,** The dark spots circled by green lines with spot size of 100~300 nm are identified as low response regions (amplitude signal strength < 45 pm, barely above the noise level.) that occupy about 15% area of the image **b**, including the 1.9% red area in image a.

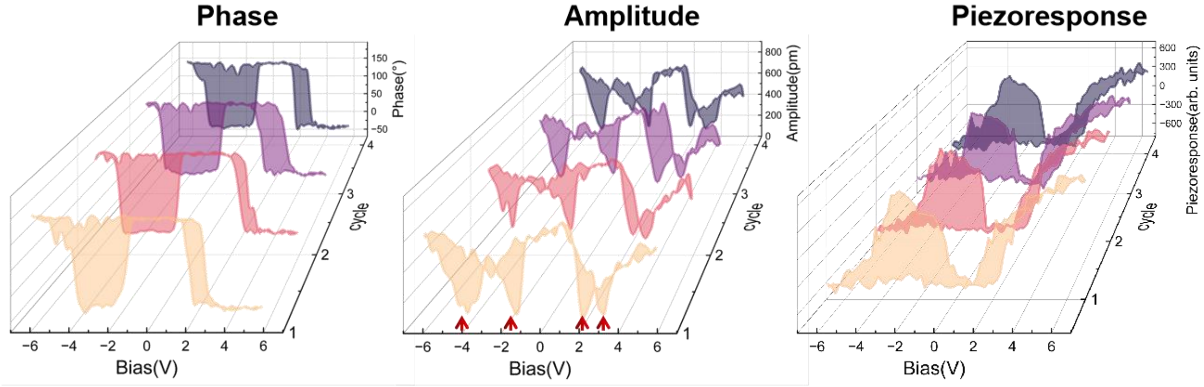

Fig. S6 | Phase and amplitude hysteresis loops, as well as the piezoresponse loops with double-subloop, showed quadruple polarization states in a CIPS film.

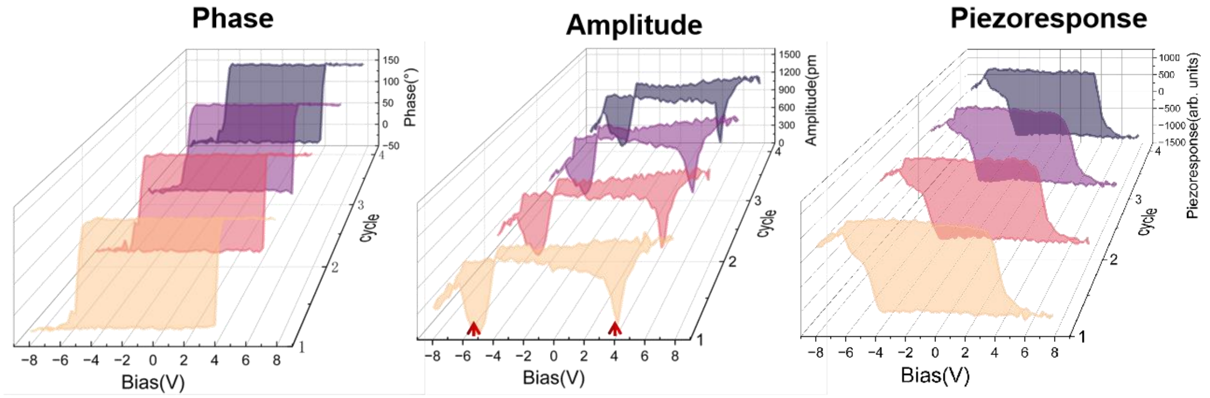

Fig. S7 | Regular bistable ferroelectric phase and amplitude hysteresis loops, as well as the piezoresponse loops, demonstrated double polarization states in a CIPS film.

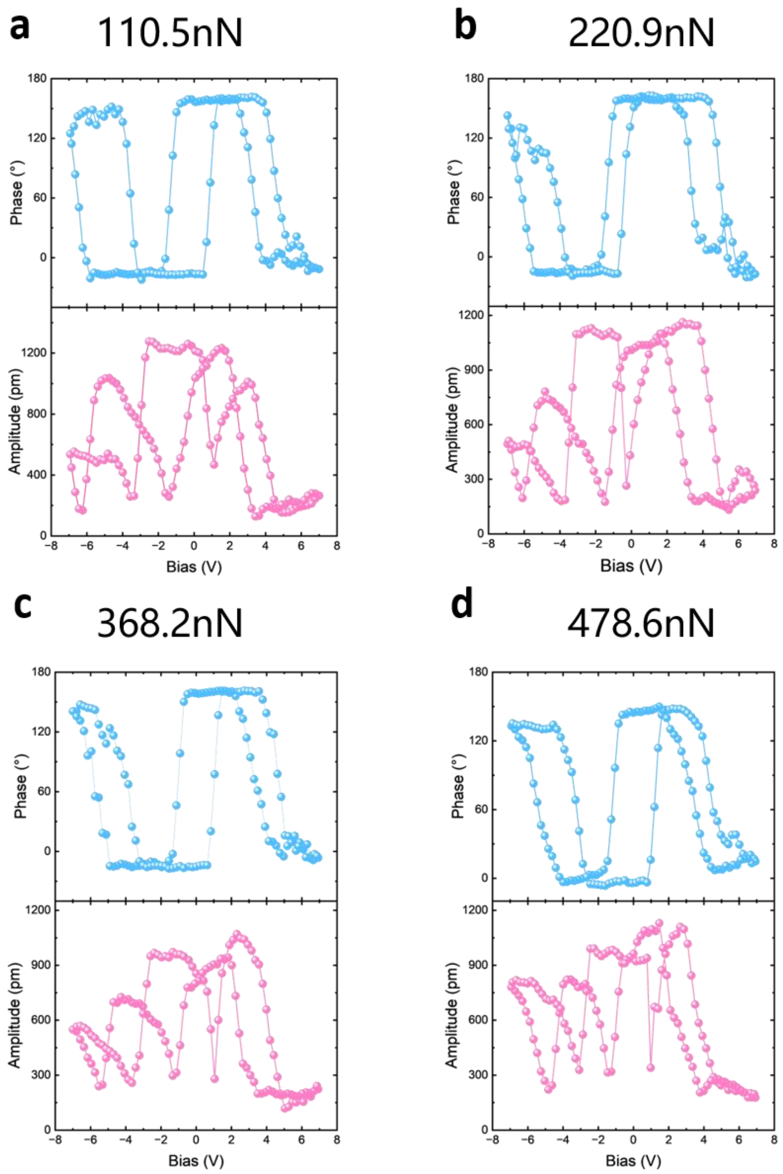

Fig. S8 | Loading force-dependent SS-PFM hysteresis loops measured at the same location. **a-d,** The gradually increasing loading forces (110.5 ~ 478.6 nN) were applied by the PFM tip in the OOP direction during the measurements of hysteresis loops and the results showed that the number of switchable polarization states did not change, only the loop shape altered slightly.

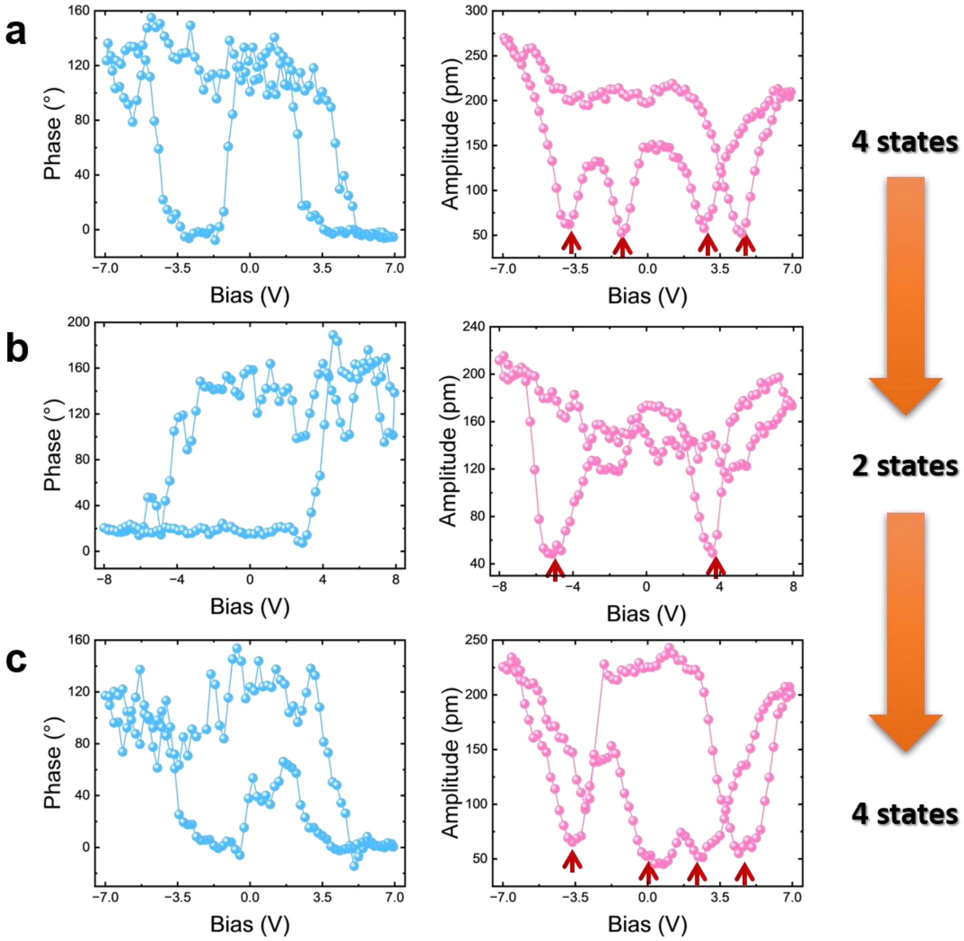

Fig. S9 | Mutual transformation between the orders of quadruple- and double-polarization states observed at the same location in a CIPS film that originally displayed quadruple polarization states. **a,** Initially observed quadruple polarization states under a bias window of ±7 V_{dc}. **b,** Observed double polarization states when a bias window was increased ±8 V_{dc}. **c,** Quadruple polarization states were recovered when a bias window was reduced to ±7 V_{dc}.

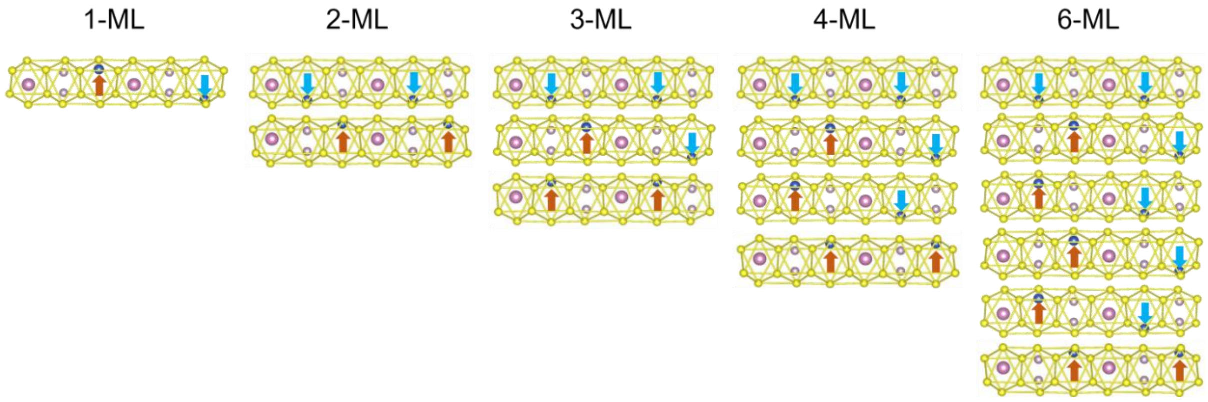

Fig. S10 | Calculated ground states of CIPS films based on DFT (1-, 2-, 3-, and 4-ML) and machine learning (6-ML).

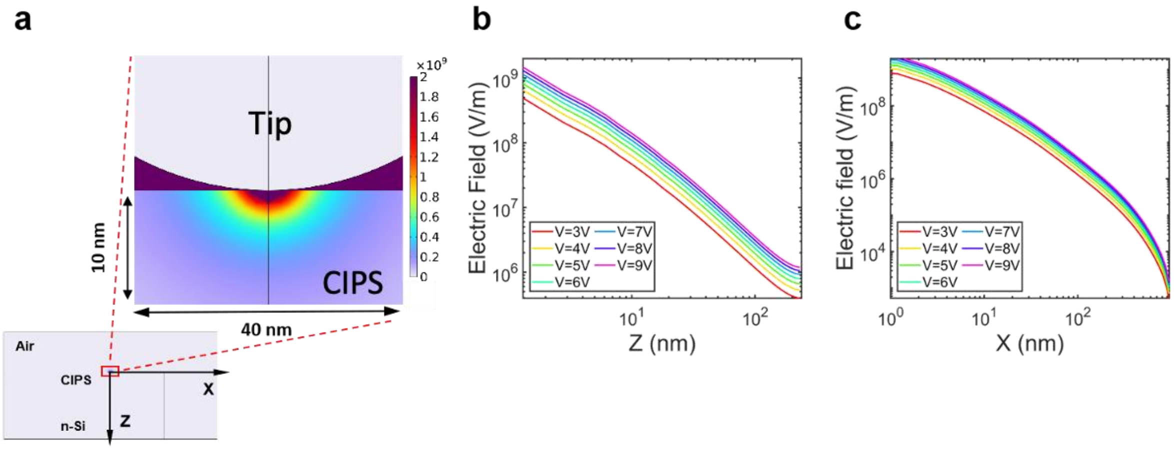

Fig. S11 | Simulated electric field distribution inside the CIPS film under different tip voltages. a, Electric field distribution when the CIPS film thickness was set to 238.7 nm and the tip voltage was set to 9 V_{dc}. **b,** Variation of the electric field intensity along the out-of-plane Z direction with respect to the tip-sample contact as the origin under different tip voltages. **c,** Variation of the electric field intensity along the in-plane X direction with respect to the tip-sample contact as the origin under different tip voltages. The modeled E-field is nearly exponentially decayed in both Z- and X-directions away from the tip-sample contact.

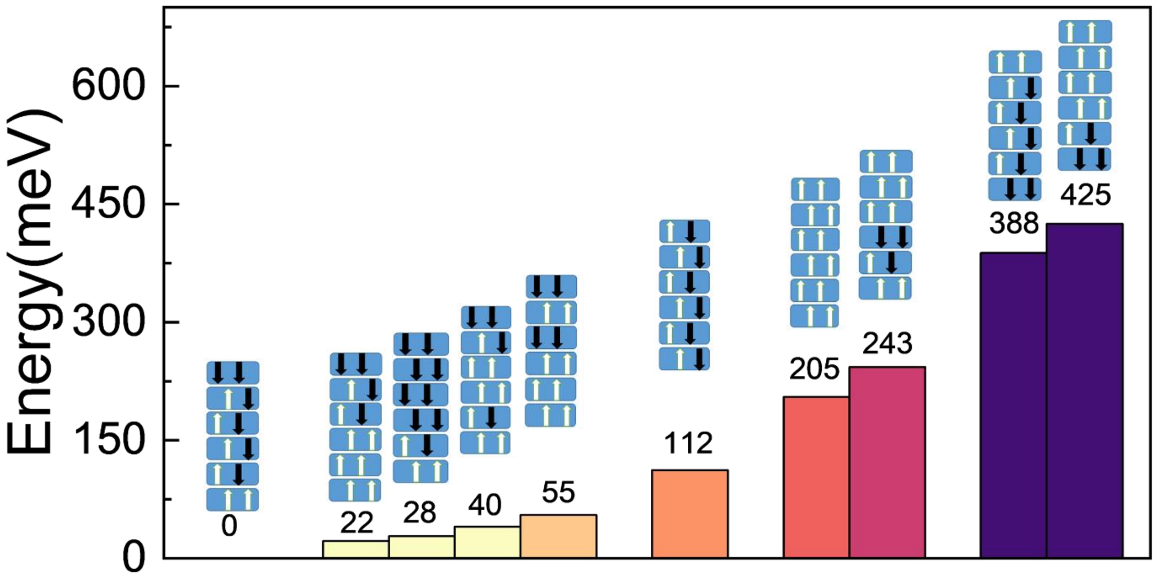

Fig. S12 | Example of several polarization configurations with different relative energy levels for the 6-ML CIPS. The ground state is an overall AFE state. It is clear that the AFE-coupled top and bottom FE MLs always exist in the configurations with lower energy (≤ 55 meV), making it an energy-favorable configuration. Comparing the ground state (0 meV) to the configuration with all AFE MLs (112 meV), the only difference is whether the top and bottom MLs are interlayer AFE-coupled FE MLs or both are AFE MLs. It indicates that the Cu ions away from the top and bottom surfaces of the film are highly desirable to maintain a lower total energy level. Once the head-to-head configuration of the outer MLs is changed to head-to-tail (≥ 205 meV) or tail-to-tail (≥ 388 meV), the total energy is further increased significantly. Our additional calculations found that this trend is also seen for much thicker films, e.g., 10-ML CIPS has the identical ground state as 3-, 4-, and 6-ML CIPS, and the total energy for the state with all AFE MLs is 112 meV, and for the state with all FE MLs is 219 meV.

State	S-1	S-4	S-2	S-5	S-3	S-6
Piezoresponse level (exp)	2.93	2.48	2.35	2.25	0.69	1.29
	LH-1 (S-1 & S-4)		LH-2 (S-2 & S-5)		LL-1	LL-2
Average value	2.71		2.30		0.69	1.29
Ratio to LH-1 (exp)	1.00		0.85		0.25	0.48
Ratio to LH-1 (hybrid mechanism)	1.00		0.83		0.25	0.50

Table S2 | Comparison of the piezoresponse level ratios observed by experiments and those calculated based on the hybrid mechanism. The normalized absolute values of the experimentally observed piezoresponse levels of S-1~S-6 are shown in the first row of the table. Taking LH-1 as the reference level, the ratios of piezoresponse levels of LH-2, LL-1, and LL-2 to LH-1 were observed to be 0.85, 0.25, and 0.48, respectively, close to the ratios calculated from the hybrid mechanism (the last row) (Fig. S15).

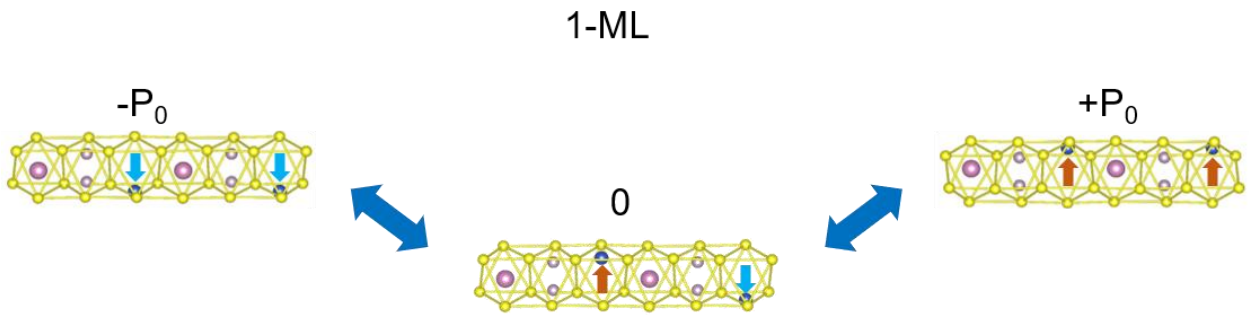

Fig. S13 | Schematic of the layer-selective half-layer-by-half-layer switching proposed in the AFE/FE model[5].

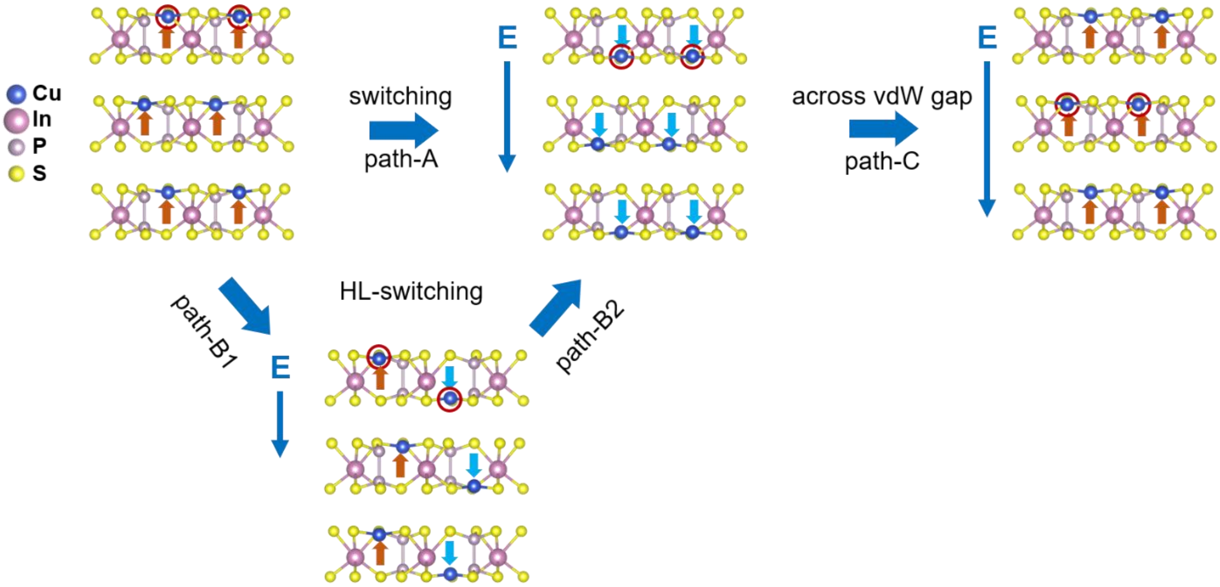

Fig. S14 | Illustrations of different types of polarization switching paths. Regular polarization switching paths through path-A, movements of Cu ions during "half-layer-by-half-layer" switching (HL-switching) through path-B1 and path-B2, and unusual polarization switching (polarization direction opposite to E-field) caused by Cu ions migration across the vdW gaps through path-C. The movements of two example Cu ions are tracked in red circles to demonstrate the different types of switching.

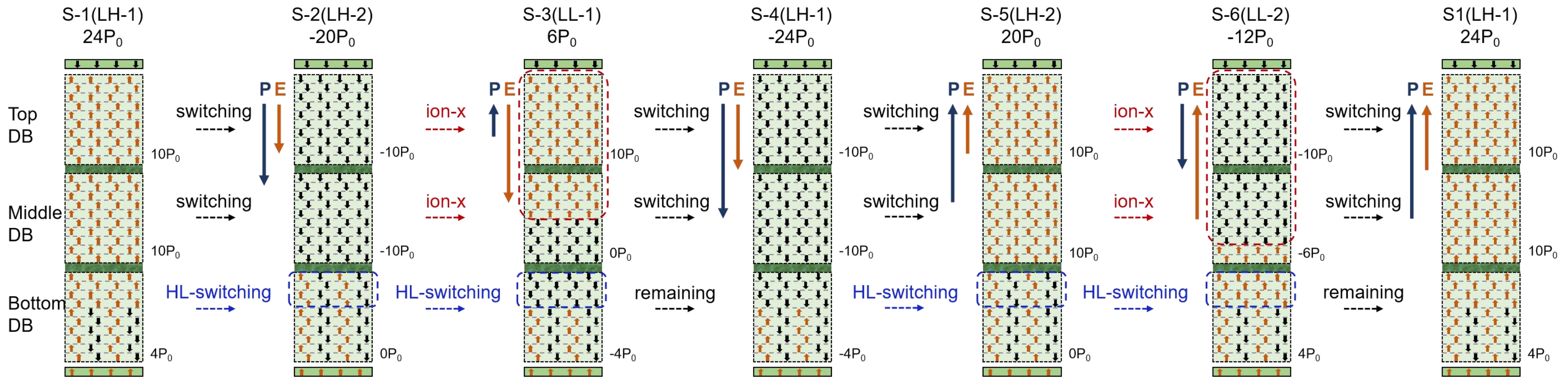

Fig. S15 | A probable detailed configuration of three DBs that form sextuple polarization states during a polarization switching cycle. P_0 is defined as the polarization value of an FE ML. Taking the total polarization of LH-1 ($24P_0$) as the reference level, the ratios of piezoresponse levels of LH-2 ($20P_0$), LL-1 ($6P_0$), and LL-2 ($12P_0$) to LH-1 were calculated to be 0.83, 0.25, and 0.50, akin to the experimentally observed piezoresponse levels (Table S2 and Fig. 5b). Here "switching" indicates the regular polarization switching process, "HL-switching" indicates the half-layer-by-half-layer switching process, and "ion-x" indicates the process involving Cu ions migration crossing vdW gap.

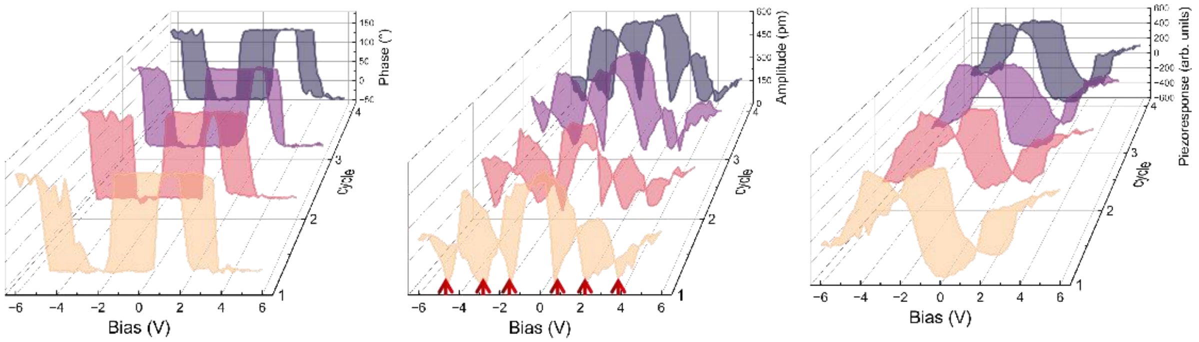

Fig. S16 | Additional dataset showing clear six minima in multiple cycles of amplitude loop.

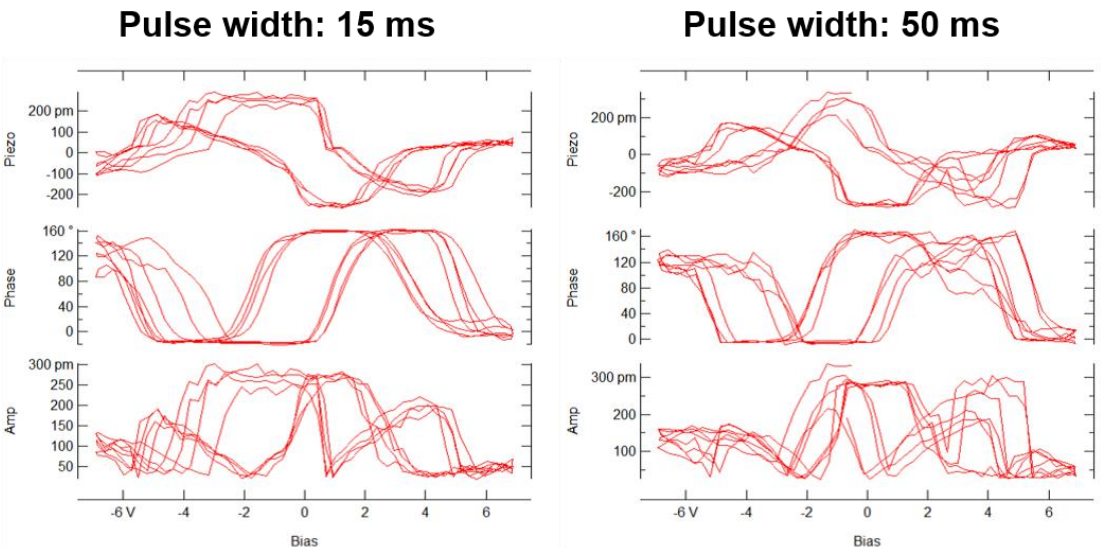

Fig. S17 | Ferroelectric hysteresis loops demonstrating sextuple polarization states obtained under pulse widths of 15 and 50 ms. The top row shows the piezoresponse, the middle row shows the phase signal, and the bottom row shows the amplitude signal.

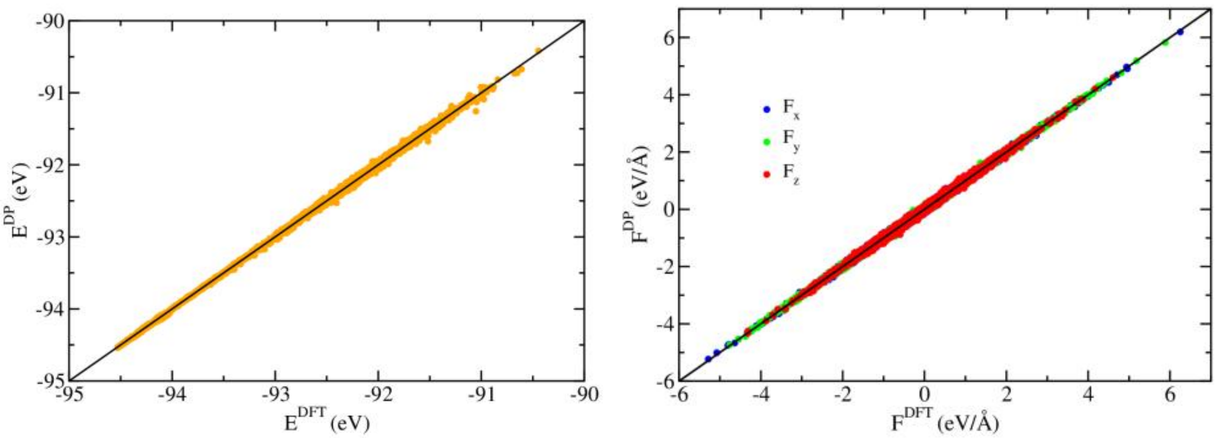

Fig. S18 | Comparison of energies and atomic forces predicted by DFT and DP model for all configurations in the test dataset.

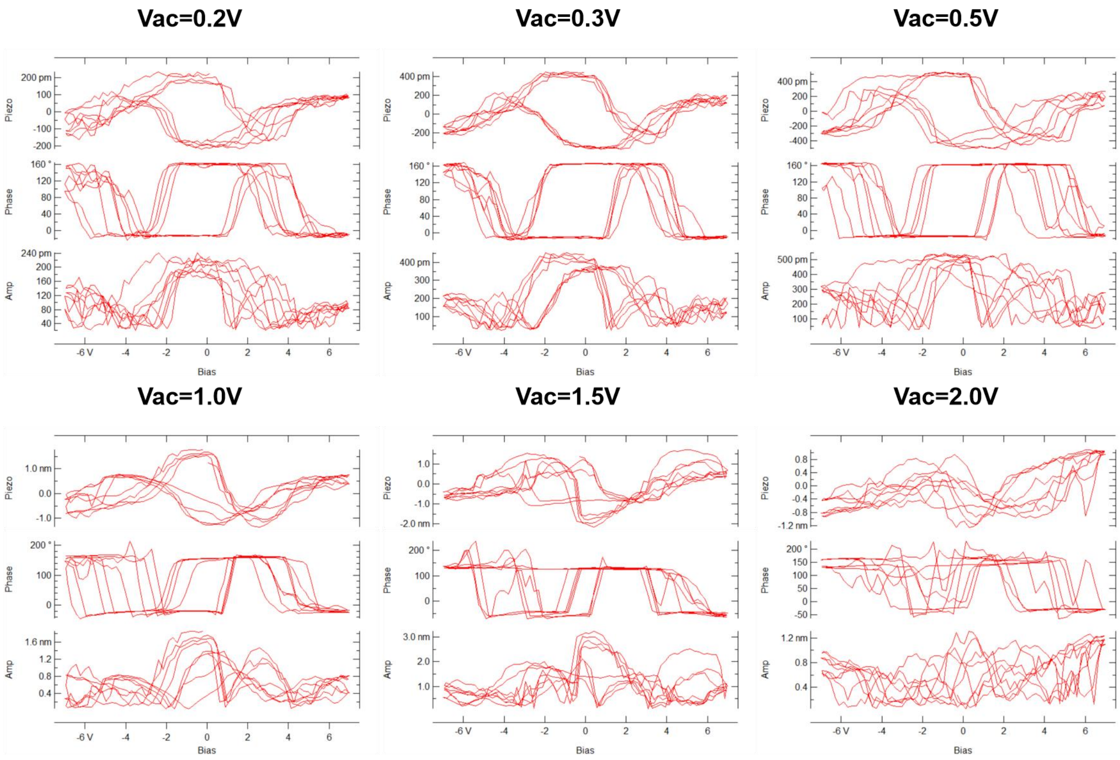

Fig. S19 | Effect of varied detection voltage V_{ac} on the hysteresis loop of sextuple polarization states. For each panel, the top, middle, and bottom rows show the piezoresponse, phase, and amplitude signals, respectively.

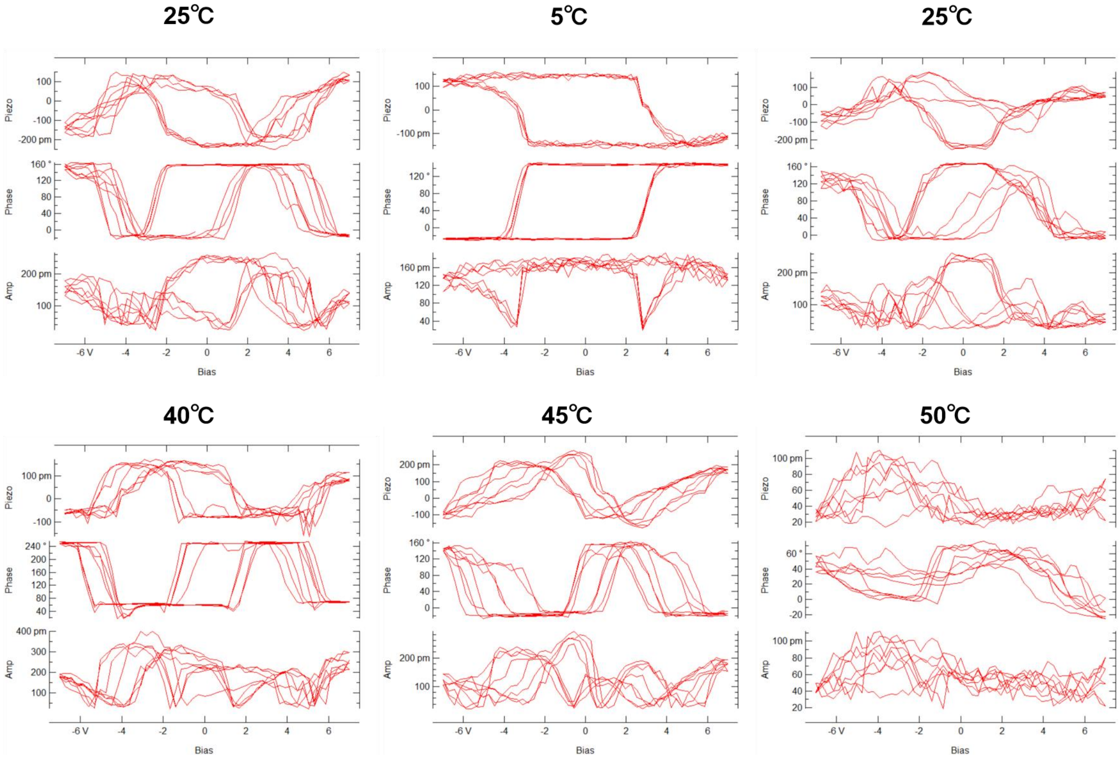

Fig. S20 | Temperature-dependent ferroelectric hysteresis loops. For each panel, the top, middle, and bottom rows show the piezoresponse, phase, and amplitude signals, respectively.

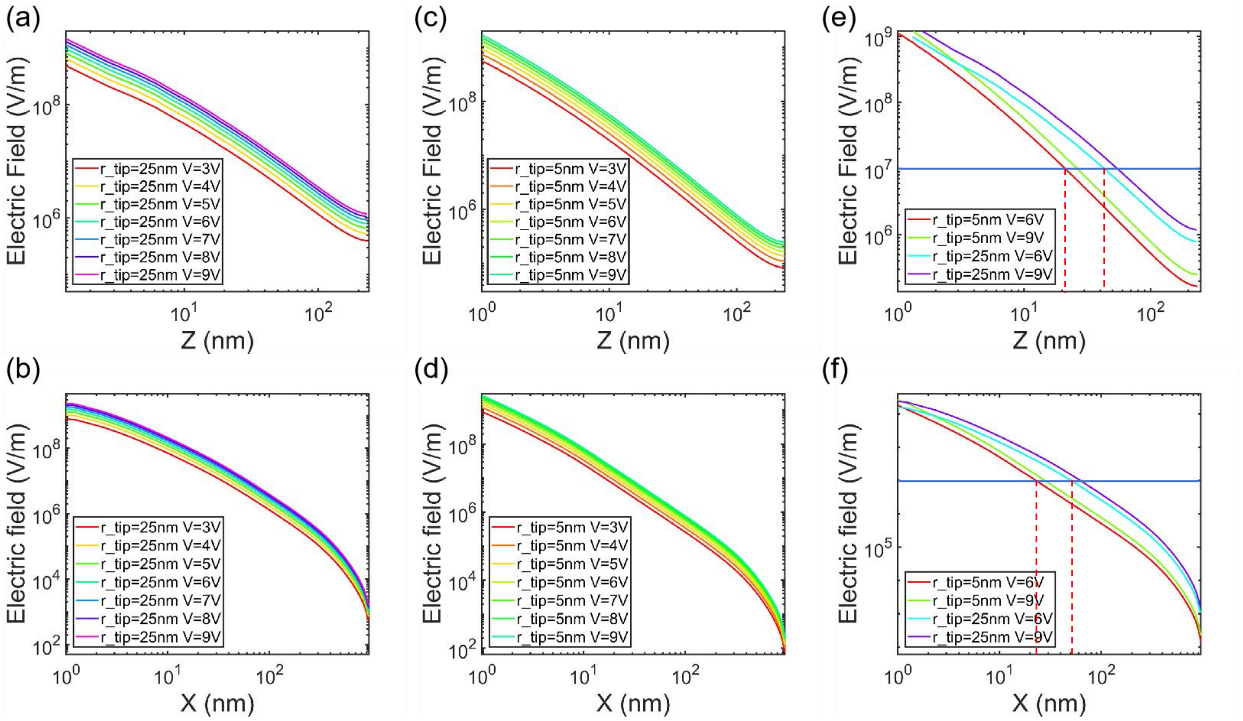

Fig. S21 | Electric field distribution in CIPS under a conductive tip of 5 nm radius. (**a,c,e**) Electric field distribution along the depth direction (Z). (**b,d,f**) Electric field distribution along the in-plane direction (X).

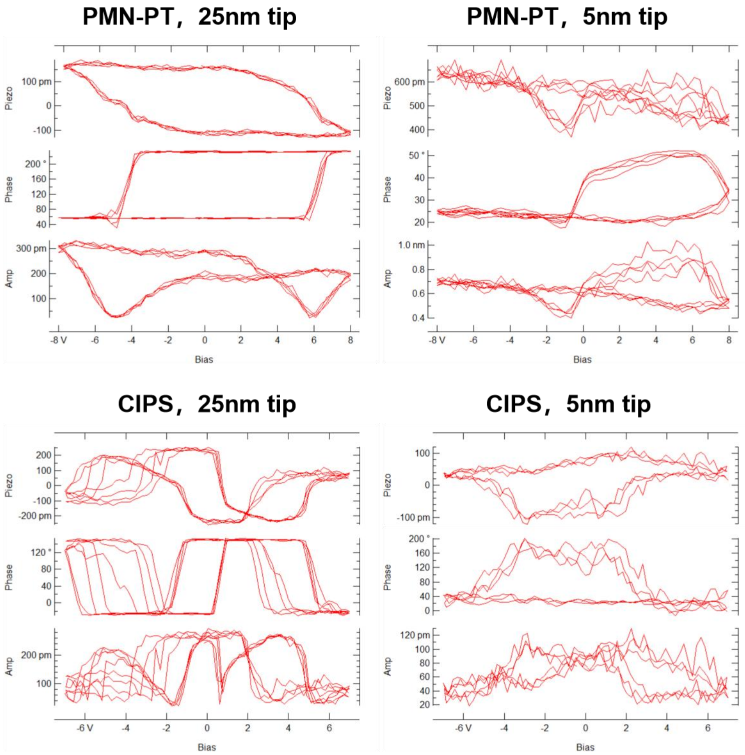

Fig. S22 | PFM ferroelectric loops of PMN-PT and CIPS measured using conductive tips with ~25 nm and ~5 nm radius, respectively.

	-Vc3	-Vc2	-Vc1	+Vc1	+Vc2	+Vc3
Average value from one sample	-5.42	-3.10	-1.76	0.74	3.28	4.90
Standard deviation from *one* sample (std1)	0.37	0.54	0.32	0.11	0.61	0.60
Average value from multiple samples (Fig. 2e)	-6.13	-3.71	-2.02	1.21	3.88	5.87
Standard deviation from *multiple* samples (std2)	1.10	0.96	0.59	0.63	1.08	1.29
Ratio of std1/std2	0.34	0.56	0.54	0.17	0.56	0.47

Table S3 | Comparison of the average and the standard deviation of coercive voltages measured from one sample and multiple samples showing sextuple polarization states.

References

1 Neumayer, S. M. *et al.* Alignment of polarization against an electric field in van der Waals ferroelectrics. *Phys. Rev. Appl.* **13**, 064063 (2020).

2 O'Hara, A., Balke, N. & Pantelides, S. T. Unique features of polarization in ferroelectric ionic conductors. *Adv. Electron. Mater.* **8**, 2100810 (2021).

3 Deng, J. *et al.* Thickness-dependent in-plane polarization and structural phase transition in van der Waals ferroelectric $CuInP_2S_6$. *Small* **16**, e1904529 (2020).

4 Zhou, S. *et al.* Anomalous polarization switching and permanent retention in a ferroelectric ionic conductor. *Mater. Horiz.* **7**, 263-274 (2020).

5 Yu, G., Pan, A., Zhang, Z. & Chen, M. Polarization multistates in antiferroelectric van der Waals materials. arXiv.2312.13856 (2023).
